# Perception of Safety and Liking Associated to the Colour Intervention of Bike Lanes: Contribution from the Behavioural Sciences to Urban Design and Wellbeing

**DOI:** 10.1371/journal.pone.0160399

**Published:** 2016-08-22

**Authors:** Pablo Vera-Villarroel, Daniela Contreras, Sebastián Lillo, Christian Beyle, Ariel Segovia, Natalia Rojo, Sandra Moreno, Francisco Oyarzo

**Affiliations:** 1 School of Psychology, Universidad de Santiago de Chile USACH, Santiago de Chile, Chile; 2 Innovation Centre in Information Technology for Social Applications (CITIAPS), Universidad de Santiago de Chile USACH, Santiago de Chile, Chile; Peking UIniversity, CHINA

## Abstract

The perception of colour and its subjective effects are key issues to designing safe and enjoyable bike lanes. This paper addresses the relationship between the colours of bike lane interventions—in particular pavement painting and intersection design—and the subjective evaluation of liking, visual saliency, and perceived safety related to such an intervention. Utilising images of three real bike lane intersections modified by software to change their colour (five in total), this study recruited 538 participants to assess their perception of all fifteen colour-design combinations. A multivariate analysis of covariance (MANCOVA) with the Bonferroni post hoc test was performed to assess the effect of the main conditions (colour and design) on the dependent variables (liking towards the intervention, level of visual saliency of the intersection, and perceived safety of the bike lane). The results showed that the colour red was more positively associated to the outcome variables, followed by yellow and blue. Additionally, it was observed that the effect of colour widely outweighs the effect of design, suggesting that the right choice and use of colour would increase the effectiveness on bike-lanes pavement interventions. Limitations and future directions are discussed.

## Introduction

For a long time cyclists have used and shared the same road as motorised vehicles, creating unsafe conditions particularly for the former, who are more vulnerable and have fewer protections [1].

The intervention of colour in the bike lane is one of the measures used to increase safety for cyclists as well as for pedestrians and motorists [2].

Colour must be considered relevant both in terms of road safety and aesthetics. Its function to promote safety corresponds to the aims of adequately indicating events or particularities of the lane, and ensuring their message is recognised correctly and that this message is located in the right place [3]. It is also important to consider the use of colour in the environment as a factor that promotes the feeling of liking among an area’s inhabitants, which is connected to the subjective meaning attributed to it [2]. In this sense urban planning acquires great relevance around the world, considering that visual quality and the environment are important factors when increasing the well-being and safety of a city’s inhabitants [4,5], and behavioural sciences are able to contribute to it [6–9].

Different countries around the world have intervened in the pavement colour of bike lanes for intersections with a heavy traffic flow and/or with higher accident rate so as to increase their safety while also creating more pleasant urban surroundings, using mainly blue, red, yellow and green [10–13]. Nevertheless, there is insufficient evidence of any colour being used more or less or the reasons that would bear out such a choice.

There are still few data on the effect of the intervention of pavement colour despite the impact this can have on road safety. Studies in this area include that of Hunter et al. [14], who used blue to mark the ten most troublesome intersections in the city of Portland, USA. Among the outcomes, modifications to certain cyclist behaviours were reported, such as turning one’s head to look for a motorised vehicle (which went from 43% to 26%) and signalling a turn (reduced from 11% to 5%), both related to the sensation of safety that this intervention provided. In addition, before painting the pavement 63% of the motorists signalled in the area of contention, a behaviour that increased to 84%, and the percentage of motorists that yielded in the affected area raised from 70% to 92%.

In 2005, the Chicago Department of Transportation [15] used green to paint the bike lanes, a decision based on previous studies that found that this colour contrasts well with the pavement and has good visibility, thus increasing safety [10–13,16].

In other lines of investigation, research has addressed the issue of the perception of colour and its correlation with subjects’ behaviour through studies that report a comprehensive analysis of the relation between colour, meaning and psychological functioning. In this research area, Fetterman [2] reviews the psychology of colours, especially red due to its particular evolutionary and adaptive connotations, which renders it a transcultural colour associated with conditions that require alertness.

However, the findings that have been obtained in this research area have not been incorporated sufficiently into government programmes. For example, a study conducted in Melbourne, Australia, assessed the effect of red ochre on bike lane demarcations. Their results showed that red is well accepted, reporting that it increased the feeling of safety for the cyclists studied, while motorists were more aware of the existence of the cyclists sharing the lanes. Nevertheless, the road authority chose to use green to paint the bike lanes, which differs with the data reported in the previously mentioned study [17].

In Santiago de Chile—where the present study was conducted—the planning of new bike lanes does not present clear indications regarding the application of a specific colour, design, or any foundations for the choice [14,15]. Currently streets in Santiago have bike lanes of various colours, including blue, yellow, red, white and green. In addition, there are three different designs applied to road intersections, namely fully-painted intersection, line-delimited intersection, and chess-board design. Colours and designs are utilized at discretion of the local authority, which could cause confusion instead of providing safety due to the lack of a uniform criterion for the road intervention [18,19].

Avant et al. had already warned of the negative consequences of the lack of signalling standardization [20]. These authors investigated the effect on attention and response times to consistent or different symbols or signals (same sign, symbol and word of the same meaning, or signs with a different meaning). They found that the latency times and the accuracy of the response were better when there was consistency between the signs than when they were different, which applied to road safety suggests that the lack of norm or standardization of bike lane colour and design can affect their effectiveness.

In summary, investigations into the use of colour in bike lanes have reported an effect of the intervention in bike lanes with colour on the accident rate, as well as on the feeling of safety and the coexistence of the different users. At the same time, these studies have shown that there are still disagreements with respect to the colour, extent and design of the intervention on the pavement for bike lanes. Under this scenario, the authorities in charge of their design have partial information of data in this area, which translates into decisions which are not always scientifically validated and a shortage of empirical guidelines to help them decide which colour and design are best to secure the objectives of increasing safety and well-being in the use of bike lanes.

In light of the foregoing, the present study proposed in general to assess the perceptions that the inhabitants of Santiago de Chile have of the colour interventions used in bike lanes. Specifically, the relationship between the colour and visual saliency of a bike lane intersection and the subjective sensation of liking and the feeling of safety that the colour produces were analysed. At the same time, the effect of three types of designs of these interventions in Santiago on the same variables (visual saliency, safety and liking) was assessed. With these results it was expected that one or more colour and design combinations could be identified that can be more effective when designing a bike lane visual intervention in order to contribute with scientifically validated data in the generation of future norms and/or guidelines for the design of urban bike lanes. The procedure and materials involved are explained next.

## Materials and Method

### Participants

The sample was composed of 538 Chilean adults, with 424 fully completed surveys (79%), while the remaining presented various degrees of completeness, therefore analyses were performed excluding cases pairwise. The gender distribution shows 53.7% were female. Age ranged from 18 up to 58 or more, with 72.9% of the sample being concentrated between 18 and 32 years. The recruitment was non-probabilistic, voluntary, and web-based through e-mailed invitations. As the target users of bike lanes is the general population, regardless of their visual condition, no exclusion criteria related to visual conditions were considered as part of the design of this study.

### Ethics statement

The study was part of a larger project on wellbeing approved by the ethics boards of Fondecyt (one of the Chilean science support offices) and the Universidad de Santiago de Chile—USACH. Informed consent was obtained explicitly from the participants as part of the electronic questionnaire used to collect the information. Participants were informed of their right to leave the study at any time if they wished without providing explanations or having some kind of consequence for them. Personal information of all participants was anonymized to keep their privacy.

### Procedure

**Fig 1:**
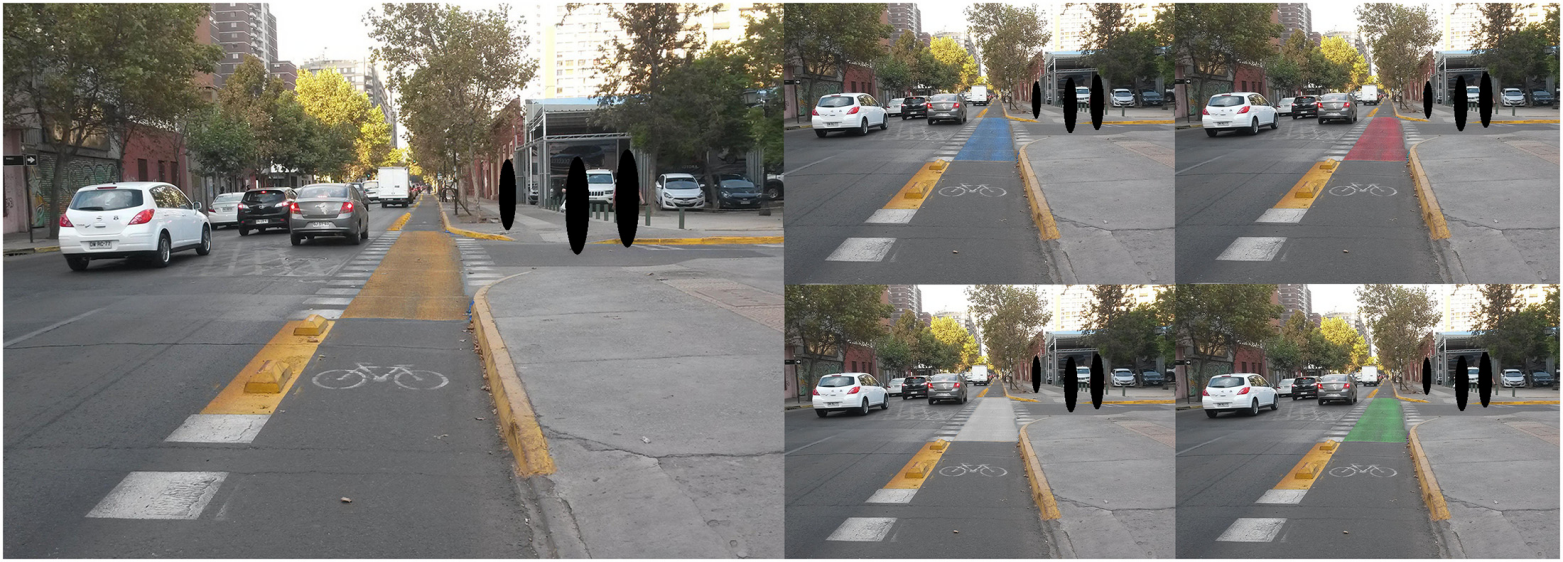
Pictures of the fully painted intersections.

**Fig 2:**
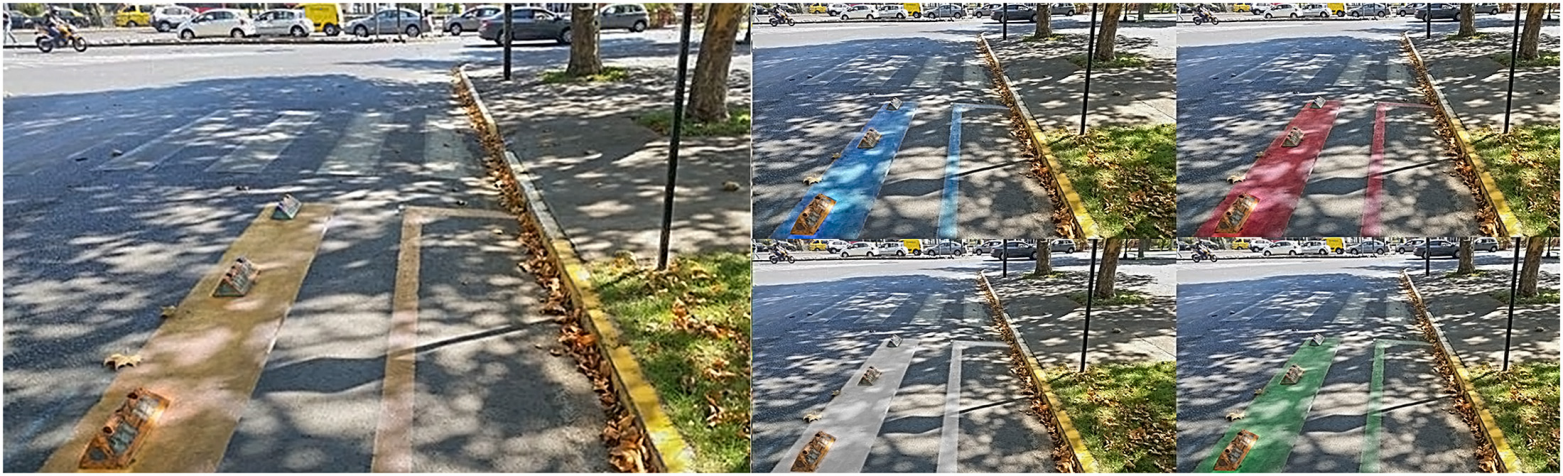
Pictures of the line-delimited intersections.

**Fig 3:**
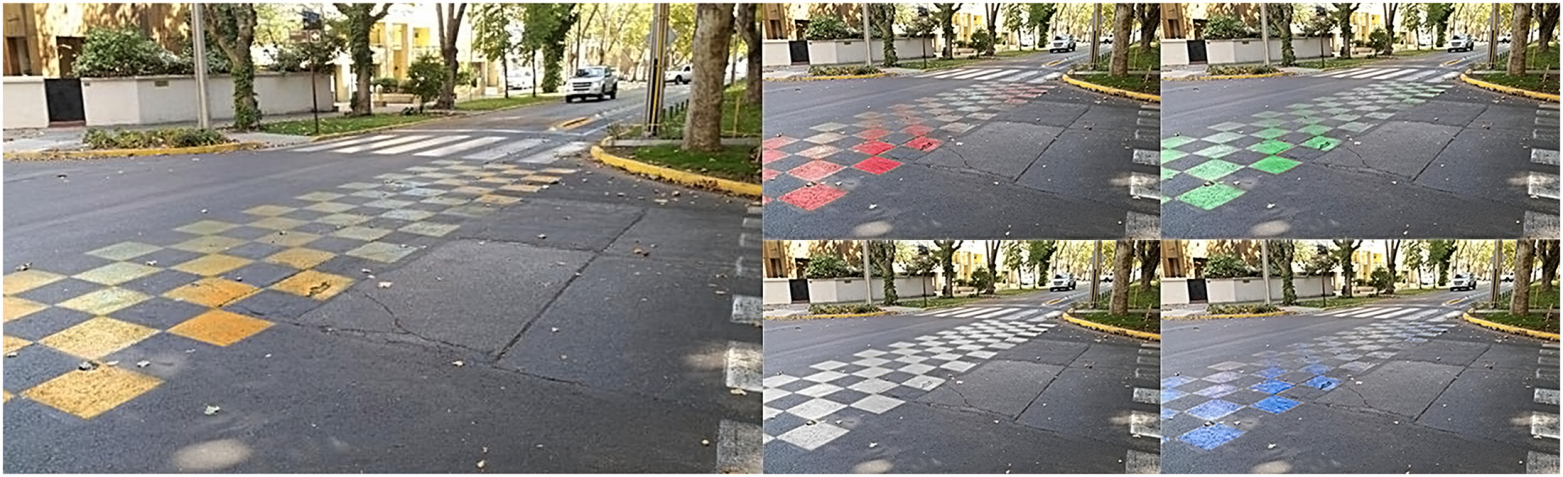
Pictures of the partially painted intersections.

The information was gathered through a computer-based quasi-experimental setting. Pictures of three real bike lane intersections were taken by our team (one fully painted intersection, one line-delimitation, and one intersection partially painted in a chess board design) and then digitally processed to modify the pavement colour intervention in order to display each design in yellow (L*a*b: 61.07, 6.39, 28.64), blue (L*a*b: 55.69, -6.08, -18.48), green (L*a*b: 55.59, -14.77, 10.23), white (L*a*b: 80.60, 0.00, 0.00) and red (L*a*b: 54.33, 34.92, 9.55). The pictures represent the three most commonly used road-intersection visual enhancements, which are shown in Figs 1 to 3.

The actual size of the previous images on screen (21 inches LCD monitor) was 20.5x11.5 cms for all the pictures. Following the display of one picture, a set of questions was presented related to perceptions of safety and aesthetic liking induced by the picture. The procedure was repeated until all 15 pictures had been randomly presented. Finally, participants were asked to complete a demographic questionnaire.

### Instruments

**Fig 4:**
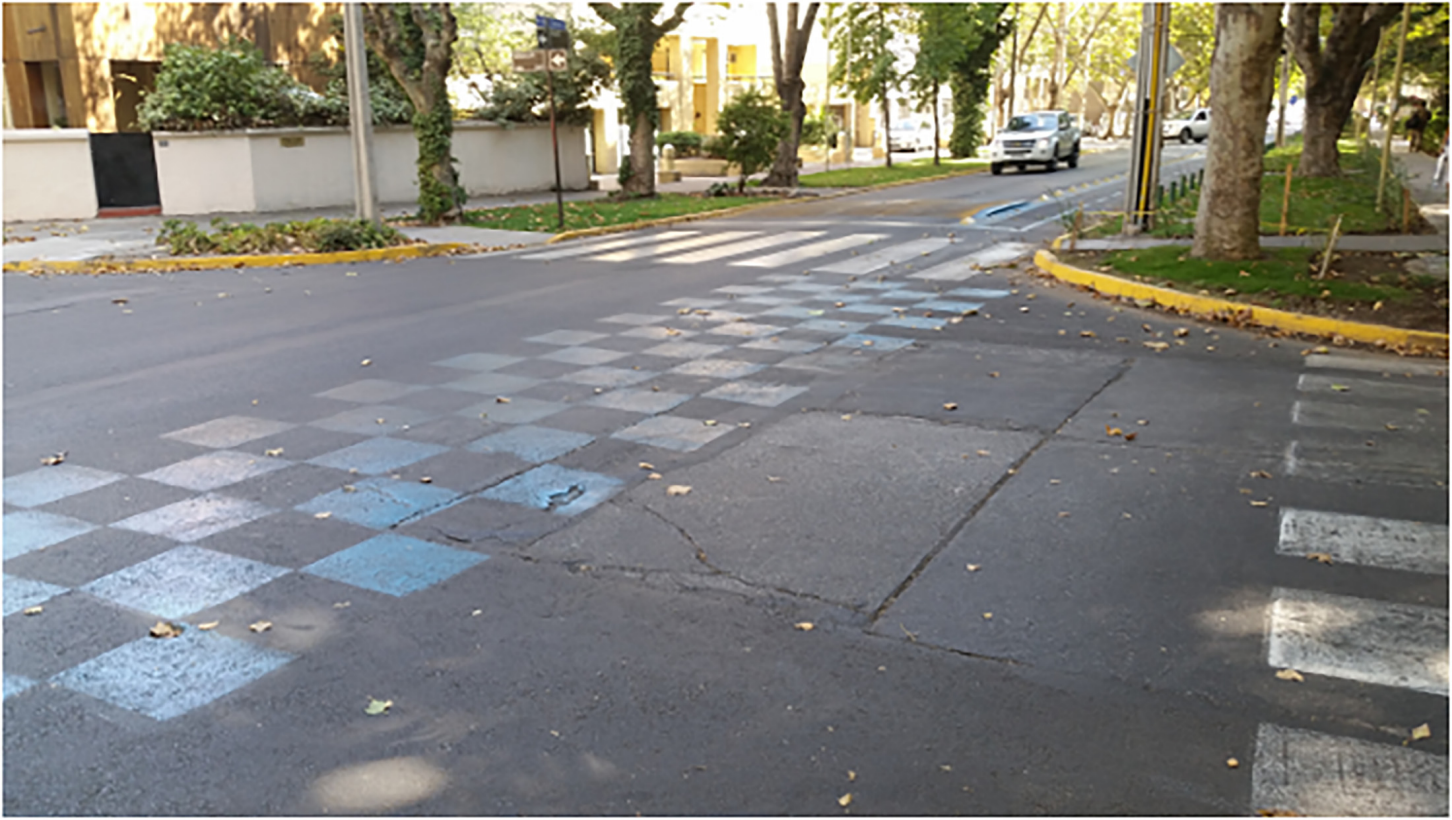
Example question. One picture of the research setting and the questions that follow it.

The data were collected utilising one set of items designed by the authors. It consisted of 3 statements about colour-liking, saliency of the bike lane intersection, and safety perception, which were presented together with each picture (15 times in total). Each statement was followed by a five-point scale of agreement (1 = I strongly disagree; 5 = I strongly agree). An example of these questions can be found below in Fig 4.

- Q1: The colour of this bike lane intersection is clearly distinguishable.
- Q2: The colour of this bike lane intersection seems pleasant to me.
- Q3: The colour of this bike lane intersection seems appropriate for my safety.

## Results

### Analytical strategy

A series of descriptive analyses were used to characterise subjects’ responses according to different conditions, combining colour and bike lane intersections. Afterwards, the mean score for each colour*design were grouped by outcome variable and compared utilising a paired samples t-test, performing a Bonferroni post hoc test to determine differences between conditions. Finally, a multivariate analysis of covariance (MANCOVA) was performed utilising SPSS v.22 to assess the effect of pavement colour and pavement design on people’s perception of liking, saliency and safety of the bike lane intersection, accompanied of a linear regression with perception of safety as dependent variable alone, as the variable more directly related to risk.

### Descriptive Analyses

**Fig 5:**
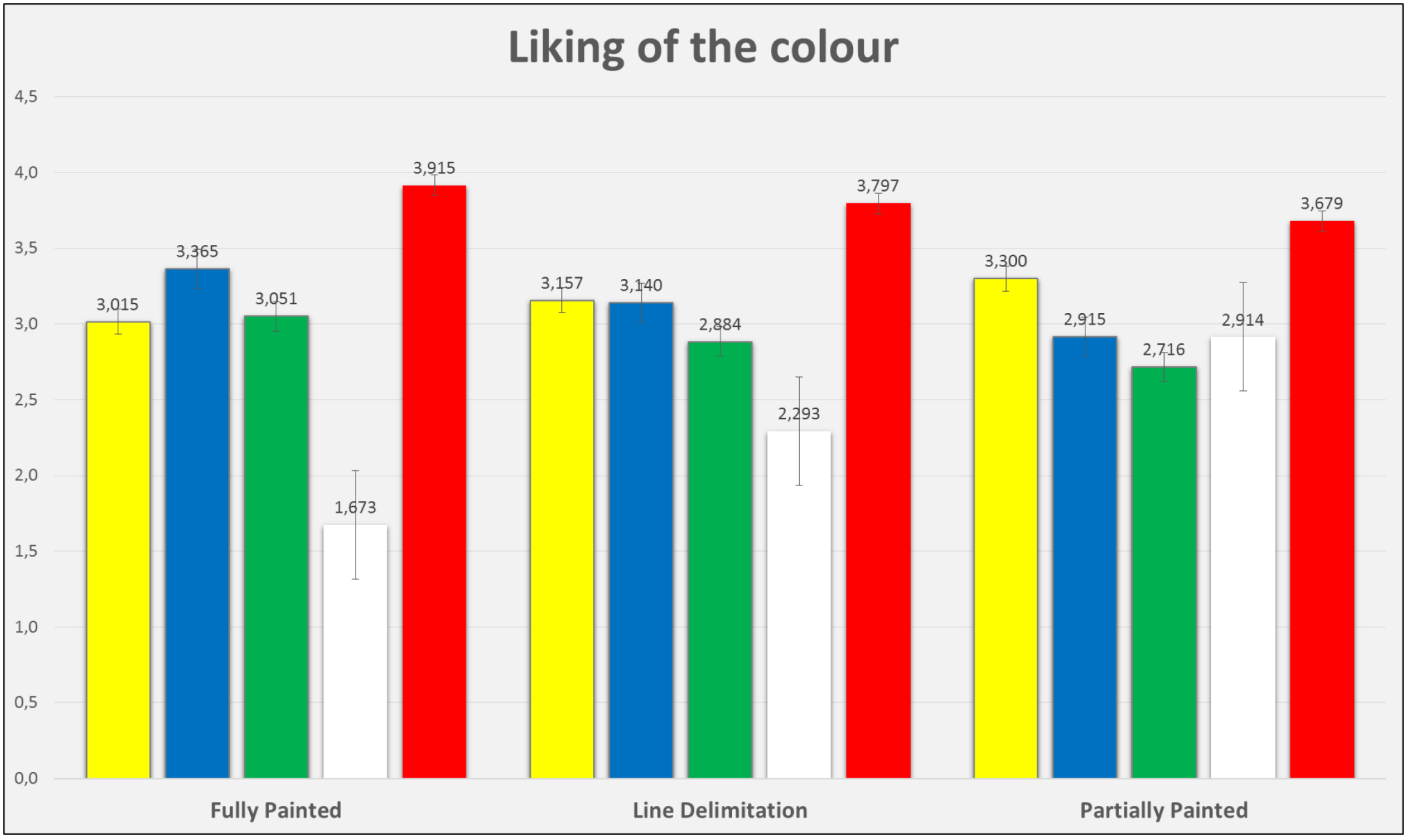
Mean scores and error bars of the variable “liking of the colour”, graphically represented for each colour and the three bike lane intersections.

**Fig 6:**
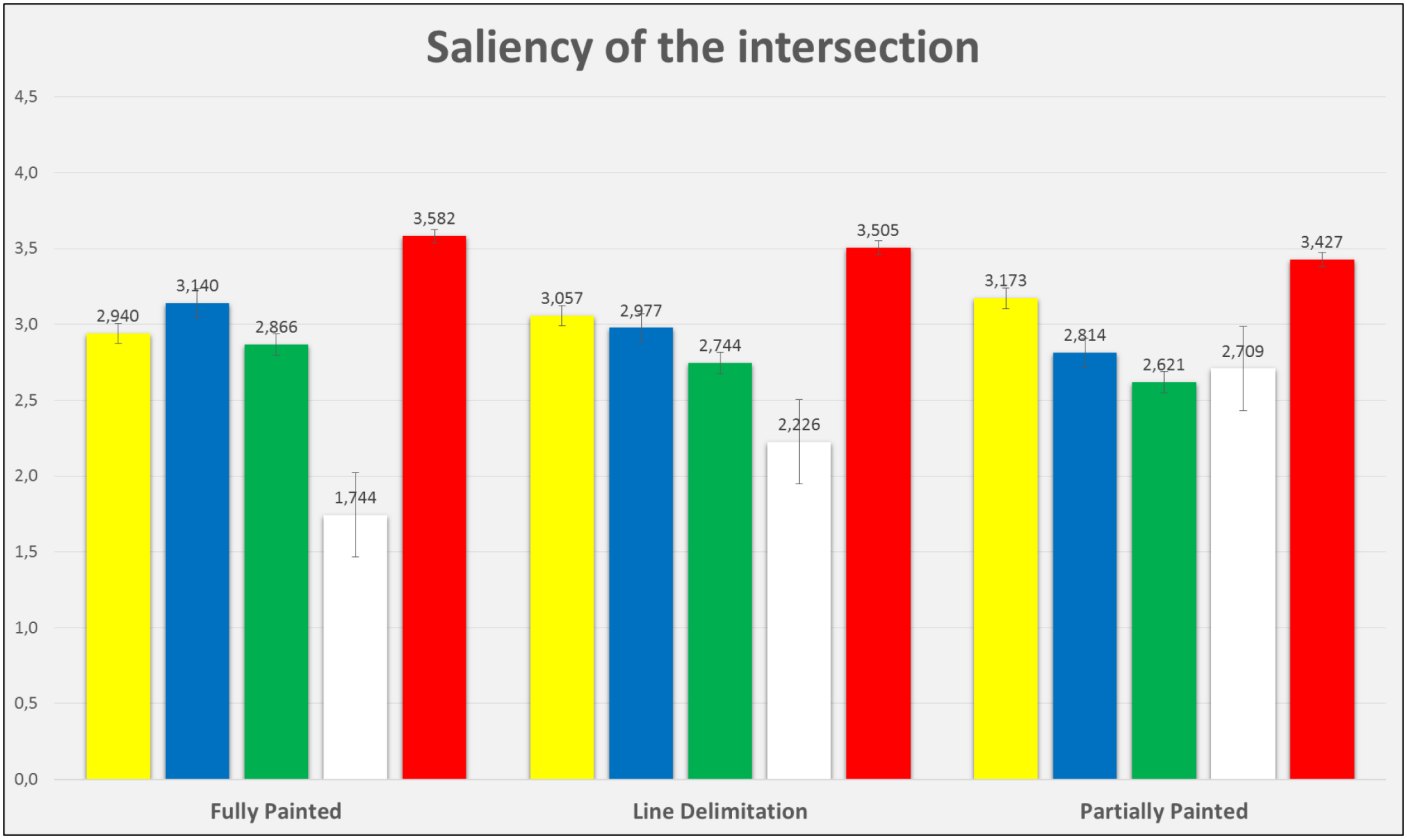
Mean scores and error bars of the variable “saliency of the intersection”, graphically represented for each colour and the three bike lane intersections.

**Fig 7:**
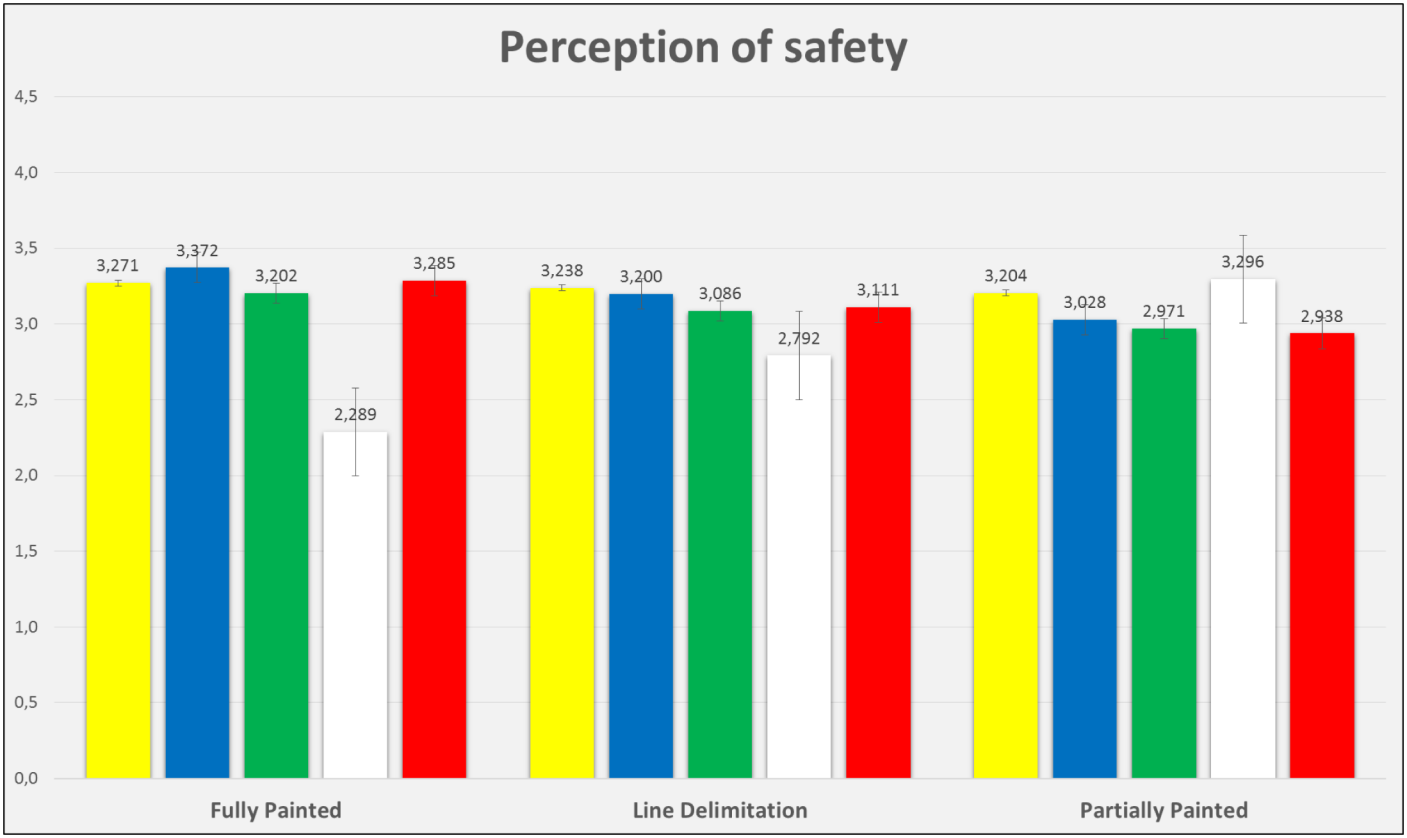
Mean scores and error bars of the variable “perception of safety”, graphically represented for each colour and the three bike lane intersections.

Figs 5 to 7 show the mean scores of participants’ liking for the colour, the evaluation of the pavement design saliency and the perception of safety. Such results are displayed by colour and intersection design, and it is possible to appreciate that their values are similar, with the exception of white, which presents lower mean scores for most of the conditions.

The relationships between the outcome variables can be observed in Table 1. There is a significant correlation between them, and a particularly high one for the pair liking-saliency.

**Table 1:** Pearson correlations between dependent variables.

Correlations			
Pearson correlation	Liking	Saliency	Safety
Liking	1.000		
Saliency	0.822**	1.000	
Safety	0.428**	0.476**	1.00

** The correlation is significant at p<0.01 (bilateral).

We wanted to assess the differences in the scores obtained in Likeness, Saliency and Safety among the different colour and design settings. A t-test for dependent samples was performed for each of the dependent variables, showing statistical differences among conditions. Table 2 shows the summary of the intra-subjects effects—no sphericity assumed according to Mauchly test.

**Table 2:** Results of the intra-subject effect paired samples t-test among conditions and grouped by saliency, liking and safety.

Intra-subject effect test with Greenhouse-Geisser correction						
Saliency						
Condition	Type III sum of squares	df	Mean Square	F	Sig.	Partial Eta square
Fully-painted	1054,349	3,886	271,302	284,016	,000	0,382
Error	1707,651	1787,679	,955			
Line-demarked	883,372	3,522	250,793	225,146	,000	0,335
Error	1753,828	1574,474	1,114			
Partially-painted	200,898	3,361	59,775	50,478	,000	0,107
Error	1683,501887	1421,669	1,184			
Liking						
Fully-painted	352,446	3,591	98,154	97,114	0,000	0,187
Error	1535,154	1518,881	1,011			
Line-demarked	44,365	3,193	13,894	10,566	0,000	0,024
Error	1776,035	1350,644	1,315			
Partially-painted	42,192	3,314	12,731	11,100	0,000	0,026
Error	1607,808	1401,904	1,147			
Safety						
Fully-painted	711,894	3,849	184,940	198,214	0,000	0,301
Error	1655,706	1774,537	,933			
Line-demarked	632,458	3,539	178,721	160,855	0,000	0,265
Error	1757,542	1581,841	1,111			
Partially-painted	164,153	3,466	47,362	44,534	0,000	0,095
Error	1570,247	1476,478	1,064			

The Bonferroni post hoc test compared the mean scores among colours (I-J) and designs (Fully painted, line-delimited, and partially-painted) grouped by dependent variable, confirming that in general red has significantly higher mean scores among conditions, followed by blue. White is the one with the lowest scores between conditions, except in the condition safety/partially painted intersection. More details can be found in the summary shown in Table 3.

**Table 3:** Summary of the Bonferroni post hoc test for comparison between conditions.

Pairwise Comparison			Fully Painted Intersection	Line Delimited Intersection	Partially Painted Intersection
Dependent Variable	I	J	Mean differences (I-J)	Mean differences (I-J)	Mean differences (I-J)
Liking	Yellow	Blue	-0.351*	0.017	0.385*
		Green	-0.037	0.274*	0.584*
		White	1.342*	0.864*	0.386*
		Red	-0.901*	-0.640*	-0.379*
	Blue	Yellow	0.351*	-0.017	-0.385*
		Green	0.314*	0.256*	0.199*
		White	1.692*	0.847*	0.001
		Red	-0.550*	-0.657*	-0.765*
	Green	Yellow	0.037	-0.274*	-0.584*
		Blue	-0.314*	-0.256*	-0.199*
		White	1.378*	0.590*	-0.197*
		Red	-0.864*	-0.914*	-0.963*
	White	Yellow	-1.342*	-0.864*	-0.386*
		Blue	-1.692*	-0.847*	-0.001
		Green	-1.378*	-0.590*	0.197*
		Red	-2.242*	-1.504*	-0.766*
	Red	Yellow	0.901*	0.640*	0.379*
		Blue	0.550*	0.657*	0.765*
		Green	0.864*	0.914*	0.963*
		White	2.242*	1.504*	0.766*
Saliency	Yellow	Blue	-0.200*	0.079	0.359*
		Green	0.073	0.313*	0.552*
		White	1.196*	0.830*	0.465*
		Red	-0.643*	-0.448*	-0.254*
	Blue	Yellow	0.200*	-0.079	-0.359*
		Green	0.274*	0.233*	0.193*
		White	1.396*	0.751*	0.106
		Red	-0.442*	-0.528*	-0.613*
	Green	Yellow	-0.073	-0.313*	-0.552*
		Blue	-0.274*	-0.233*	-0.193*
		White	1.122*	0.517*	-0.088
		Red	-0.716*	-0.761*	-0.806*
	White	Yellow	-1.196*	-0.830*	-0.465*
		Blue	-1.396*	-0.751*	-0.106
		Green	-1.122*	-0.517*	0.088
		Red	-1.838*	-1.279*	-0.719*
	Red	Yellow	0.643*	0.448*	0.254*
		Blue	0.442*	0.528*	0.613*
		Green	0.716*	0.761*	0.806*
		White	1.838*	1.279*	0.719*
Safety	Yellow	Blue	-0.101	0.038	0.177*
		Green	0.069	0.151*	0.233*
		White	0.982*	0.445*	-0.092
		Red	-0.014	0.126*	0.266*
	Blue	Yellow	0.101	-0.038	-0.177*
		Green	0.170*	0.113*	0.057
		White	1.083*	0.407*	-0.268*
		Red	0.087	0.088	0.090
	Green	Yellow	-0.069	-0.151*	-0.233*
		Blue	-0.170*	-0.113*	-0.057
		White	0.913*	0.294*	-0.325*
		Red	-0.083	-0.025	0.033
	White	Yellow	-0.982*	-0.445*	0.092
		Blue	-1.083*	-0.407*	0.268*
		Green	-0.913*	-0.294*	0.325*
		Red	-0.996*	-0.319*	0.358*
	Red	Yellow	0.014	-0.126*	-0.266*
		Blue	-0.087	-0.088	-0.090
		Green	0.083	0.025	-0.033
		White	0.996*	0.319*	-0.358*

* The correlation is significant at p<0.05 (bilateral).

** The correlation is significant at p<0.01 (bilateral).

The descriptive information is useful to understand the predominance of certain colours or combinations of colours and design, but insufficient to understand the effect of the colour on the perceptions of safety, liking and visual saliency. The section below presents more analyses which will help achieve a clearer understanding of how much the colour determines some perceptions and to distinguish it from the effect of the selected intervention design.

### Multivariate Analysis

A full-factorial MANCOVA examined colour-liking, intersection-saliency and perceived safety as dependent variables, and the colour of the pavement intervention (5 categories) and the intersection design (three categories) as independent variables, with design as the covariate. It showed a significant multivariate effect for colour (F(12) = 71.713, p<0.000, Wilks’s Lambda = 0.876), intersection design (F(3) = 3.960, p<001, Wilks’s Lambda = 0.998) and the interaction between them (F(12) = 36.536, p<0.000, Wilks’s Lambda = 0.934).

The inter-subject effect tests (Table 4) shows colour as significantly affecting liking, saliency and safety. The intersection design is significantly related to liking and saliency, but not significantly related to safety. Finally, it can be observed that the interaction between colour and intersection design is significant to liking, saliency and safety, directly affecting the value of the partial eta squared (effect size). The implications are discussed in the following section.

**Table 4:** Results of the multivariate analysis of variance (MANOVA) performed to assess the effect of colour and intersection design on the dependent variables.

Tests of inter-subject effects							
Source	Dependent Variable	Type III Sum of Squares	df	Mean Square	F	Sig.	Partial Eta Squared
Corrected Model	Liking	1919.566(a)	9	213.285	178.306	0.000	0.202
	Saliency	1360.737(b)	9	151.193	133.750	0.000	0.159
	Safety	433.983(c)	9	48.220	44.481	0.000	0.059
Intercept	Liking	7919.539	1	7919.539	6620.722	0.000	0.511
	Saliency	7155.686	1	7155.686	6330.146	0.000	0.499
	Safety	8624.769	1	8624.769	7955.975	0.000	0.556
Colour	Liking	955.699	4	238.925	199.741	0.000	0.112
	Saliency	616.601	4	154.150	136.366	0.000	0.079
	Safety	389.086	4	97.272	89.729	0.000	0.054
Design	Liking	10.793	1	10.793	9.023	0.003	0.001
	Saliency	9.457	1	9.457	8.366	0.004	0.001
	Safety	0.013	1	0.013	0.012	0.911	0.000
Colour * Design	Liking	411.215	4	102.804	85.944	0.000	0.051
	Saliency	239.624	4	59.906	52.995	0.000	0.032
	Safety	277.855	4	69.464	64.077	0.000	0.039
Error	Liking	7.590.924	6346	1.196			
	Saliency	7173.608	6346	1.130			
	Safety	6879.457	6346	1.084			
Total	Liking	68791.000	6356				
	Saliency	62044.000	6356				
	Safety	67828.000	6356				
Corrected Total	Liking	9510.490	6355				
	Saliency	8534.346	6355				
	Safety	7313.440	6355				

^a^. Liking R squared = 0.202 (Adjusted R squared = 0.201)

^b^. Saliency R squared = 0.159 (Adjusted R squared = 0.158)

^c^. Safety R squared = 0.059 (Adjusted R squared = 0.058)

Given the relevance of the perception of safety to reduce the risk and improve de usage of bike lanes, an analysis of covariance (ANCOVA) was calculated to predict perception of safety, with colour and design as independent categorical variables. A significant, nonetheless small effect, was found (F(2,1) = 23.944, p<0.000), with a R square of 0.007, B estimate for colour of -0.066 (p<0.000) and for design of 0.002 (p>0.88). These results are very similar to those observed in Table 4.

Summarising, the main post-analysis highlights are the following:

1. From the colours included in the study, red is the one with the highest association to perceived liking and perceived saliency of the intersection.
2. Of the colours included in the study, white is the least consistent in generating positive perceptions.
3. There is a high correlation between how clearly people perceive the colour and the how much they like it.
4. The effect of the colour intervention on the bike lane is more relevant than the effect of the design used.

## Discussion

Many countries have decided to intervene in the colour of bike lane pavement to increase the feeling of safety and liking when people use them. However, the authorities responsible for such interventions do not have a body of systematic scientific evidence available on which to base their choice of colour and design. The goal of this research was to contribute with data regarding the effect of the colour on people’s perceptions of liking of the bike lane, visual saliency of the bike lane intersection and the attribution of safety to the bike lane. At the same time, the design of such interventions was taken into account and included in the analysis.

The main conclusion that can be inferred from the results is that the use of colour is more relevant that the design utilised, according with their effect size. Colour in the bike lanes would have a positive effect on people perceptions, especially on likeness and saliency. The effect of design is significant, but with a lower explanatory power, which might suggest equivalent effects on people’s perception despite the different designs implemented.

Nonetheless, not all the colours produce the same effect. Of the five colours included in this study—based on these being the five colours used in most pavement colour interventions—red was consistently related to higher scores of the outcome variables, which may be related to its evolutionary and adaptive role mentioned by Fetterman et al. [2]. Yellow and blue produced similar effect as well, but slightly lower. White was the weakest colour, except when the intersection was partially painted—like a chess board. There is no clear explanation for this, but it might be due to the fact that such an intervention is wider than the rest and more similar to a regular pedestrian crossing, producing better recognition and perception of safety than the rest of the colours.

Another interesting finding is the relationship between visual saliency and liking (r = 0.822, p<0.001). The design of the present research cannot answer whether visual saliency produces liking, or it is the individual’s colour preference that produces better recognition, but it is an interesting issue to pursue. The relationship between visual saliency and safety was also positive and significant (r = 0.476), a result that could be expected given the rationale that more effective signalling should induce a greater feeling of safety, but at the same time it draws even more attention to the strong relationship between visual saliency and liking. Future research should delve more deeply into these relationships, including in their design objective measures of safety to be contrasted with the subjective perceptions included in this study, and concepts such as “happy routes” [21,22] in order to assess the effect of urban design on people’s election of routes and its impact on physical and psychological wellbeing.

In summary, it can be said that the present research answered the questions that guided it. On the one hand, it endeavoured to identify the role of colour and the design of the pavement intervention in bike lanes. Based on these results, it can be stated that the use of colour is more relevant than the design chosen. It is not the same as saying that the design has no effect, but it means that the difference among designs is not statistically relevant. On the other hand, and considering the colour, it is possible to suggest some guidelines to help decide the best colour to use in a pavement intervention. From our results, red appears to be the best choice to induce liking towards the bike lane, saliency towards the marked intersection, and a sensation of subjective safety for the user. Closely behind are yellow and blue.

Regarding the limitations of the present research, it is important to highlight that no objective measures of safety were included in the design. To assess the real effect of colours in reducing the accident rate, together with the subjective experience of the users, it is relevant to make correct decisions and evaluate the convenience of the use of one colour above the others. Another limitation was the lack of validated instruments as part of the design, which was in part due to the lack of similar research to use as a reference. Nonetheless, the use of technology to design and simulate the experimental settings can be considered a suitable solution for this. The questions were clear, direct and reflected in their answer options the continuum of agreement on these variables that can be found in reality.

It could be argued that the use of pictures of real scenarios and its later modification might rest validity to the results. It was intended to replicate natural contexts as similar as possible with the techniques currently available, in order to assess situations that otherwise can be considered as highly difficult to handle and control. Furthermore, the use of pictures to elicit subjects responses has been proved useful in similar studies [23–25]. Another issue to be addressed on the use of images in research is the one related to image visualization and monitor calibration. In this study, the resolution of the 21” HP Compaq Pro 4300 monitor utilized was 1600x900 pixels, 32 bits real colour, a screen refresh ratio of 60 Hz, 100/100 brightness, under artificial light conditions (cold light incandescent bulb) without direct reflection on the screen. In order to ensure replicability L*a*b values have been also indicated.

Finally, in future research it would be interesting and relevant to have more detail in the particular points of view of cyclists, drivers and pedestrians. It can be possible to identify perceptual and attitudinal differences, and also common issues regarding road safety, aspects that the current study cannot offer.

With all of the above, it is expected that this study will be useful for those responsible for making decisions about the design of bike lanes, to other researchers in the field of colour perception and its applications, and for the general public interested in topics related to the design of a safer and nicer city to live in.

## Supporting Information

S1 Fig Pictures of the fully painted intersections. (TIF)

S2 Fig Pictures of the line-delimited intersections. (TIF)

S3 Fig Pictures of the partially painted intersections. (TIF)

S4 Fig Example question. One picture of the research setting and the questions that follow it.
(TIF)

S5 Fig Mean scores and error bars of the variable “liking of the colour”, graphically represented for each colour and the three bike lane intersections. (TIF)

S6 Fig Mean scores and error bars of the variable “saliency of the intersection”, graphically represented for each colour and the three bike lane intersections. (TIF)

S7 Fig Mean scores and error bars of the variable “perception of safety”, graphically represented for each colour and the three bike lane intersections. (TIF)

## References

[1] Vanparijs J, Int L, Meeusen R, de Geus B. Measurement in bicycle safety analysis: A review of the literature. J Accid Anal Prev. 2015;84:9–19. 10.1016/j.aap.2015.08.007 26296182

[2] Fetterman AK, Liu T, Robinson MD. Extending Color Psychology to the Personality Realm: Interpersonal Hostility Varies by Red Preferences and Perceptual Biases. J Pers. Wiley Online Library; 2015;83(1):106–16. 10.1111/jopy.12087 PMC4085135 24393102

[3] Gao X, Hong K, Passmore P, Poladchikova L, Shaposhnikov D. Colour Vision Model-Based Approach for Segmentstion of Traffic Sings. J Image Video Process. 2008;2008:1–7.

[4] Clay G, Daniel T. Scenic landscape assesment: the effects of land management juriction on public percepction of scenic beauty. J Landsc Urban Plan. 2000;49(1–2):1–13.

[5] Bulut Z, Yilmaz H. Determination of landscape beauties through visual quality assessment method: a case study for kemaliye (Erzican/Turkey). J Environ Monit Assess. 2007;141(1):121–9. 10.1007/s10661-007-9882-0 17786575

[6] Páramo P, Sandoval-Escobar M, Jakovcevic A, Ferreiro J, Mustaca A, Jengich A, et al Assessment of environmental quality, degree of optimism, and the assignment of responsibility regarding the state of the environment in Latin America. Univ Psychol. 2015;14(2):605–18.

[7] López Miguens MJ, Álvarez González P, González Vázquez E, García Rodríguez MJ. Measures of Ecological Behavior and its Antecedents: Scales Conceptualization and Empirical Validation. Univ Psychol. 2015;14(1):189–204.

[8] Barreto I, Neme Chaves SR. Effectiveness of influence tactics on pro-environmental behavior intention. Rev Latinoam Psicol. 2014;46(2):111–6.

[9] Moyano-Diaz E, Torquato RJ, Bianchi A. Contributions to the health sciences: The risky pedestrian behavior of chileans and brazilians. Ter Psicol. 2014;32(3):227–34.

[10] Brady J, Loskorn J, Mills A, Duthie J, Machemehl R. Operationla and Safety Implications of Three Experimental Bicycle Safety Devices in Austin, TX. 2011.

[11] Furth P, Dulaski D, Bergenthal D, Brown S. More Than Sharrows: Lane-Within-A-Lane Bicycle Priority Treatments in Three U.S Cities. 2011.

[12] Davis G. Colored Bike Lane- Request to Experiment. 2005.

[13] Hunter W, Srinivasan R, Martell C. Evaluation of a green Bike Lane Weaving Area in St. Petersburgo, Florida. Final Report to Departament of Transportation. 2008.

[14] Hunter W, Harkey D, Stewart R, Birk M. Evaluation of Blue Bike-Lane Treatment in Portand, Oregon. J Transp Res Rec. 2000;00–0456:107–15.

[15] Breaking barriers to bicycling: Bicycle lanes best practices and pilot treatments. Columbus; 2005.

[16] Dill J, Monsere CM, McNeil N. Evaluation of Bike Boxes at Signalized Intersections. OTREC final report. Portland State University, PDXScholar; 2011. p. 1–127.

[17] Government of South Australia. Distinctive Coloured Pavement Bicycle Lanes. [Internet]. 2016. [cited 2016 May 8]. Available: http://www.dpti.sa.gov.au/__data/assets/pdf_file/0020/40169/DOCS_AND_FILES-2818650-v8-Traffic_Management_-_AS1742_9_-_Distictive_Coloured_Pavement_Bicycle_Lanes_-_Operational_Instruction_9_3.pdf.

[18] Ministerio de Vivienda y Urbanismo. Construcción de Ciclovías: Estándar técnico [Internet]. 2015. [cited 2016 May 8]. Available: http://www.minvu.cl/opensite_20150512124450.aspx.

[19] Ciclovías de alto estándar: Hacia un nuevo modelo de movilidad urbana enfocadas en las personas y las familias [Internet]. Santiago de Chile; 2015. Available: http://www.minvu.cl/opensite_20150512124450.aspx.

[20] Avant LL, Thieman AA, Zang AL, Hsu SY. Memory codes for traffic sign information: visual vs meaning codes. Vis Veh [Internet]. 1996;5:117–23.

[21] Quercia D, Schifanella R, Aiello LM. The shortest path to happiness: Recommending beautiful, quiet, and happy routes in the city. In: Proceedings of the 25th ACM conference on Hypertext and social media. ACM; 2014. p. 116–25.

[22] Quercia D, Pesce JP, Almeida V, Crowcroft J. Psychological maps 2.0: a web engagement enterprise starting in London. In: Proceedings of the 22nd international conference on World Wide Web. International World Wide Web Conferences Steering Committee; 2013. p. 1065–76.

[23] Van Cauwenberg J, De Bourdeaudhuij I, Clarys P, Nasar J, Salmon J, Goubert L, et al Street characteristics preferred for transportation walking among older adults: a choice-based conjoint analysis with manipulated photographs. Int J Behav Nutr Phys Act. 2016;13(1):6. 26775290 10.1186/s12966-016-0331-8 PMC4715277

[24] Cannuscio CC, Weiss EE, Fruchtman H, Schroeder J, Weiner J, Asch DA. Visual epidemiology: Photographs as tools for probing street-level etiologies. Soc Sci Med. 2009;69(4):553–64. doi: 10.1016/j.socscimed.2009.06.013 19573966 10.1016/j.socscimed.2009.06.013

[25] Duque A, López-Gómez I, Blanco I, Vázquez C. Modificación de Sesgos Cognitivos (MSC) en depresión: Una revisión crítica de nuevos procedimientos para el cambio de sesgos cognitivos. Ter Psicol. 2015;33(2):103–16.

