# Supplementary figures and images for: Perception of Safety and Liking Associated to the Colour Intervention of Bike Lanes: Contribution from the Behavioural Sciences to Urban Design and Wellbeing
Source: PLoS One. 2016 Aug 22;11(8):e0160399. doi: 10.1371/journal.pone.0160399 (PMC4993498; doi:10.1371/journal.pone.0160399)

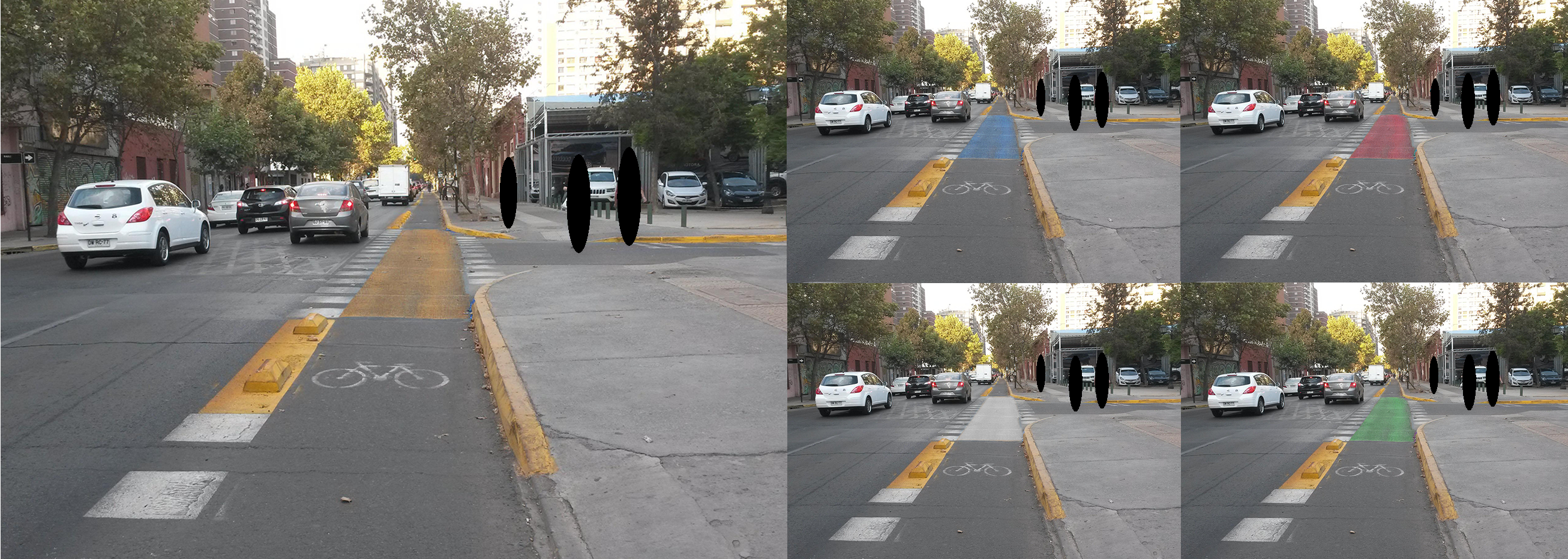

Supplement: S1 Fig — (TIF) [file pone.0160399.s001.tif]

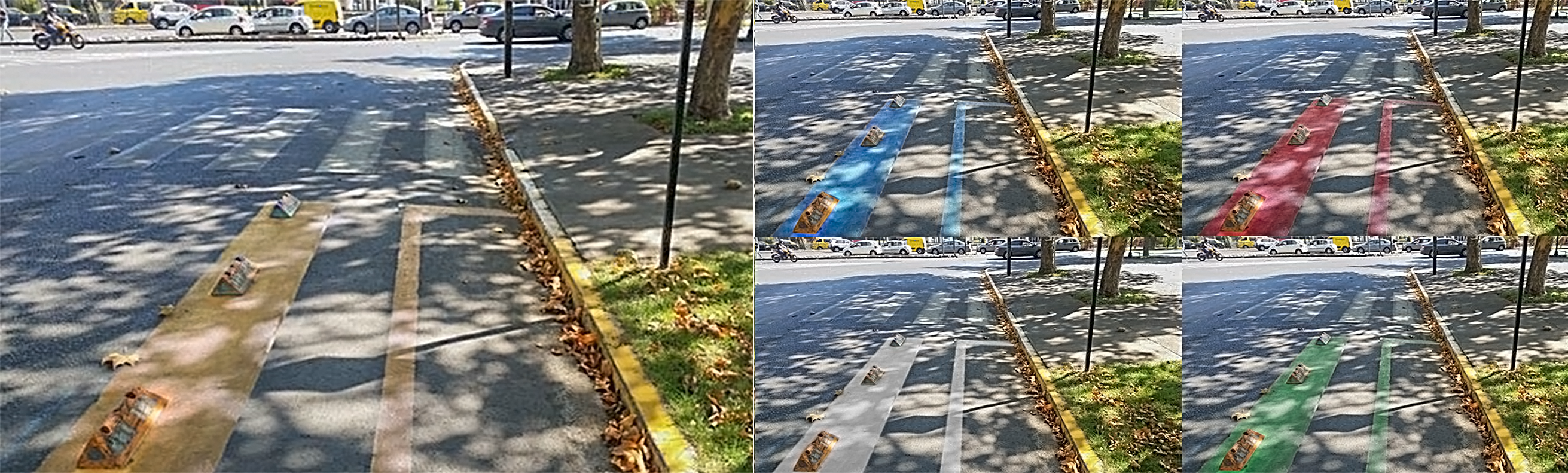

Supplement: S2 Fig — (TIF) [file pone.0160399.s002.tif]

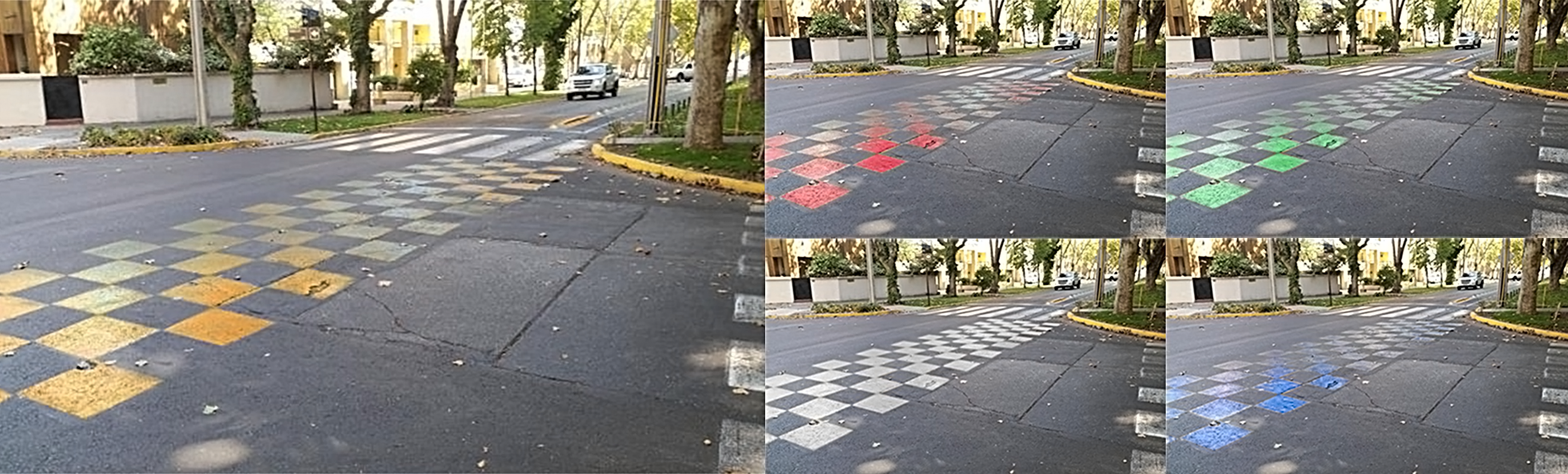

Supplement: S3 Fig — (TIF) [file pone.0160399.s003.tif]

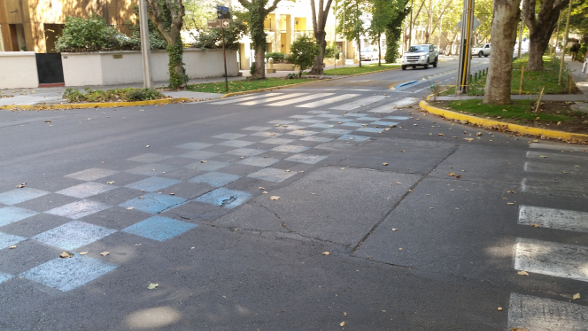

Supplement: S4 Fig — One picture of the research setting and the questions that follow it. (TIF) [file pone.0160399.s004.tif]

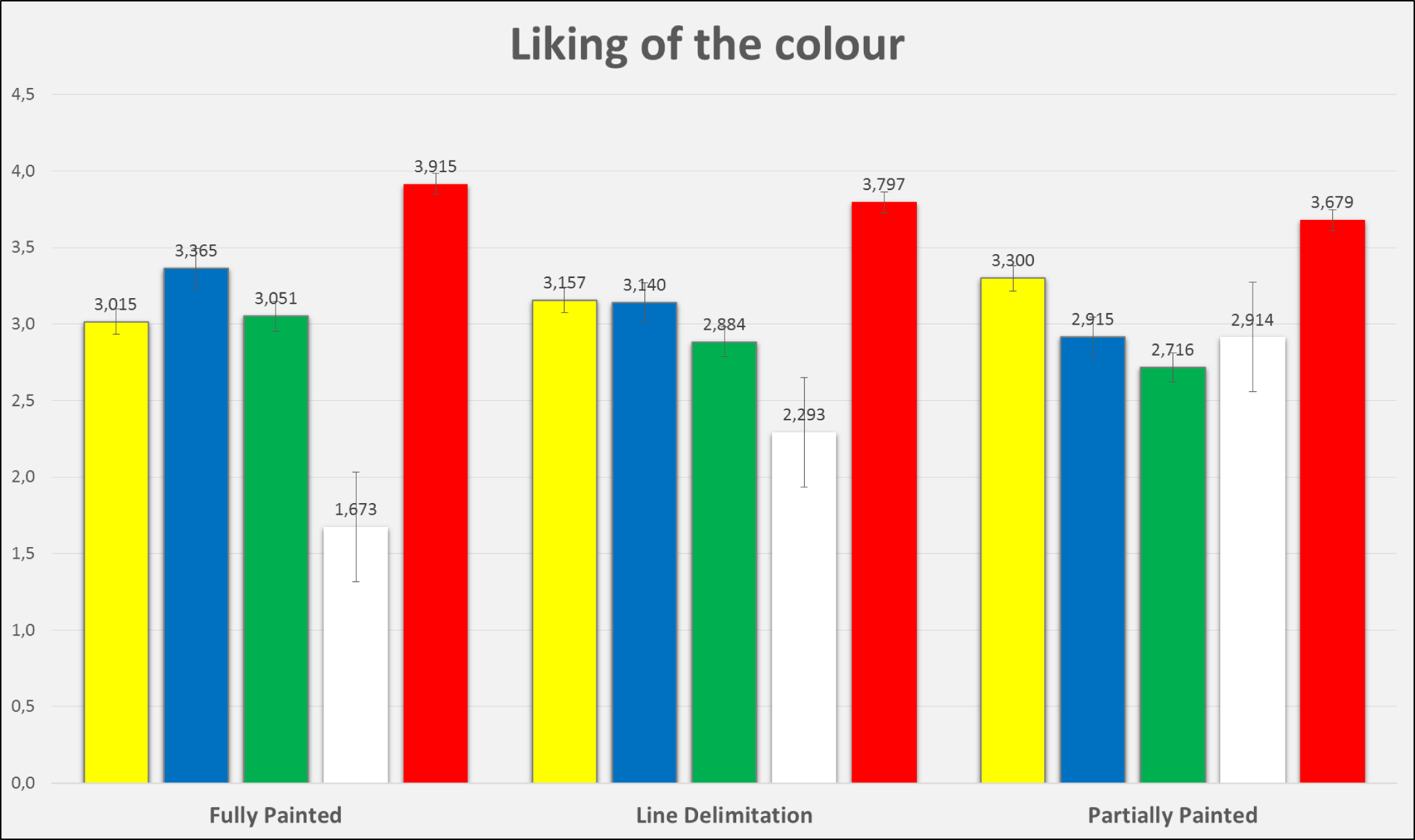

Supplement: S5 Fig — (TIF) [file pone.0160399.s005.tif]

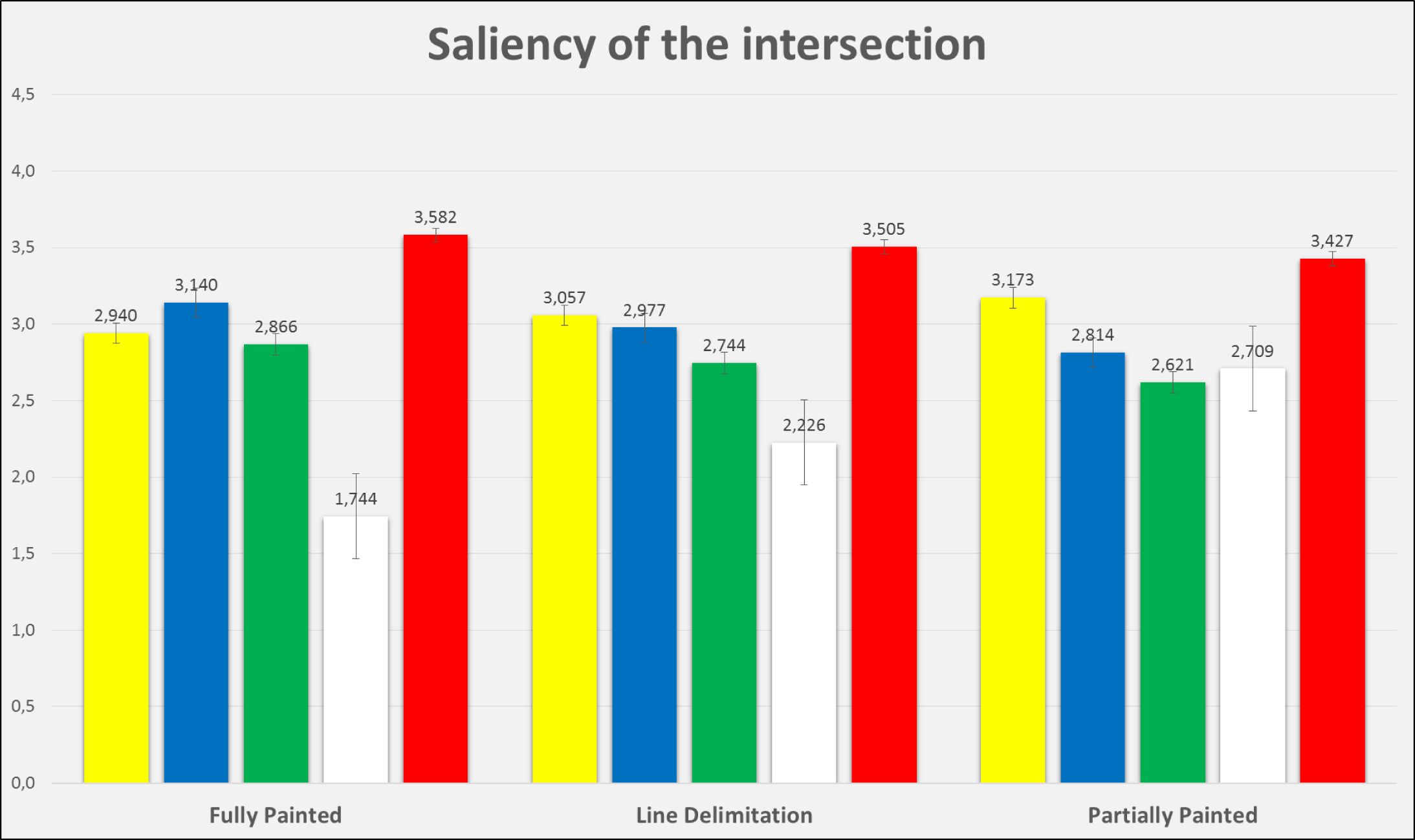

Supplement: S6 Fig — (TIF) [file pone.0160399.s006.tif]

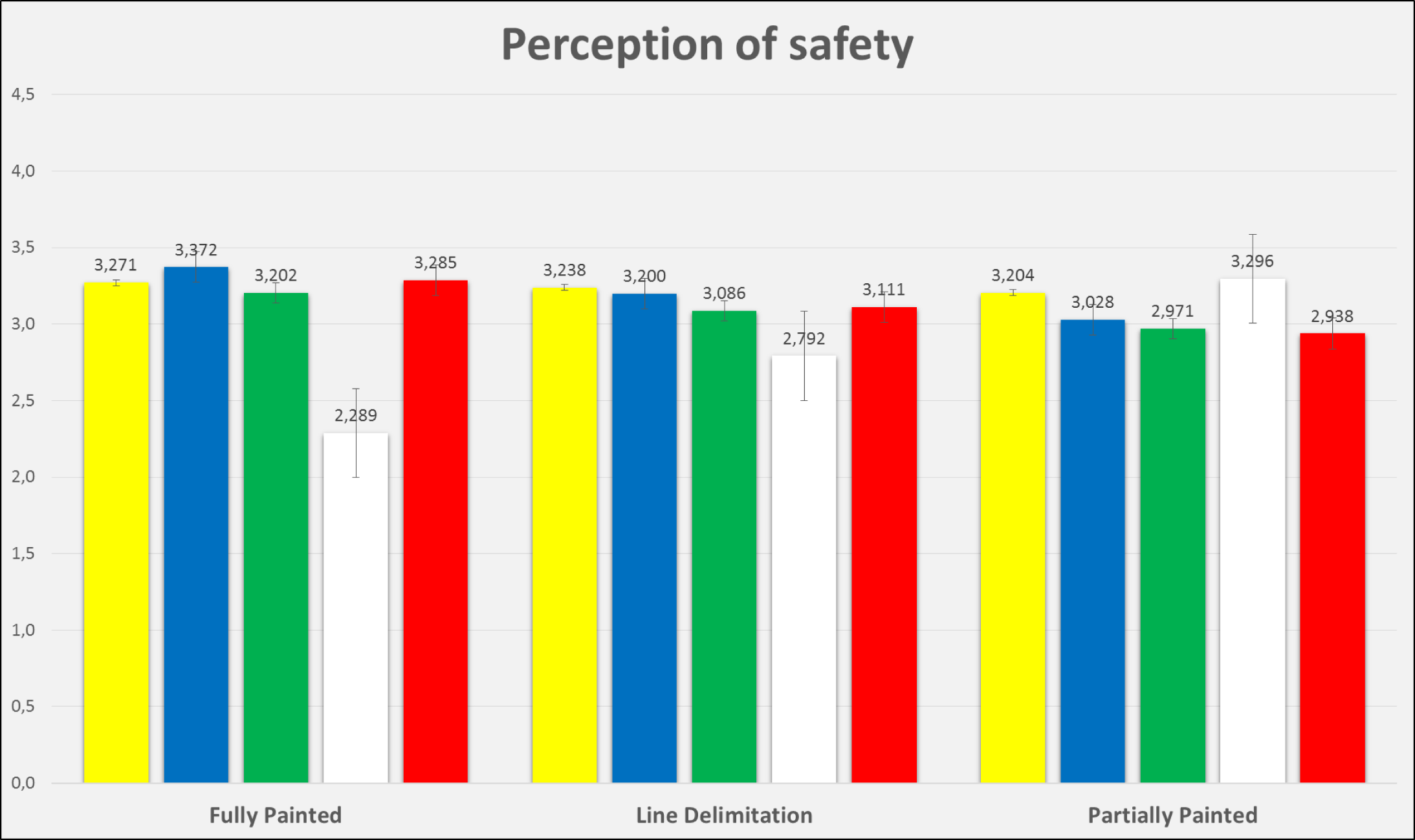

Supplement: S7 Fig — (TIF) [file pone.0160399.s007.tif]
